# P-426. Antimicrobial Resistance Focus in French ICU: A national surveillance network analysis on ventilator acquired pneumonia

**DOI:** 10.1093/ofid/ofae631.627

**Published:** 2025-01-29

**Authors:** Xavier Bourge, Arnaud Friggeri, Alain Lepape, Claire Prevot, Anais Machut, Charles-Hervé Vacheron

**Affiliations:** MSD France, VOURLES, Rhone-Alpes, France; Hospices Civils de Lyon, LYON, Rhone-Alpes, France; Centre Hospitalier Lyon Sud, Hospices Civils de Lyon, Lyon, Rhone-Alpes, France; MSD France, VOURLES, Rhone-Alpes, France; Hospices Civils de Lyon, LYON, Rhone-Alpes, France; Hospices Civils de Lyon, LYON, Rhone-Alpes, France

## Abstract

**Background:**

The aim of this study was to perform a characterization of patients who underwent mechanical ventilation, the incidence rate of ventilator acquired pneumoniae (VAP) as well as identification of the pathogens involved, their resistance profiles, and the associated clinical outcomes. This group will be scrutinized for patterns and outcomes specifically associated with ventilator use.

Cumulative incidence of VAP

**Figure:**
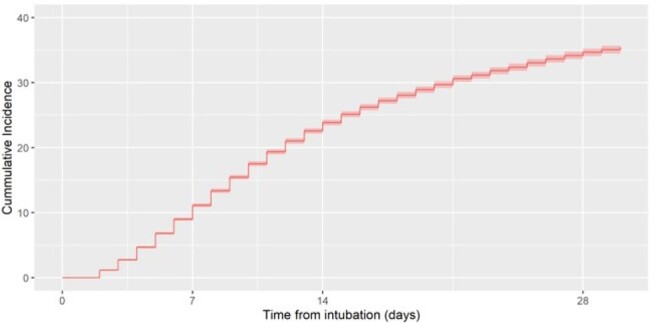

**Methods:**

Adult patients undergoing mechanical ventilation hospitalized in France for at least 48 hours in ICU participating to REA-REZO surveillance network between 2018 and 2021 were included in this cohort. To estimate the cumulative incidence of VAP, extubation and death were considered as competing event.

Description of the cohort

**Figure:**
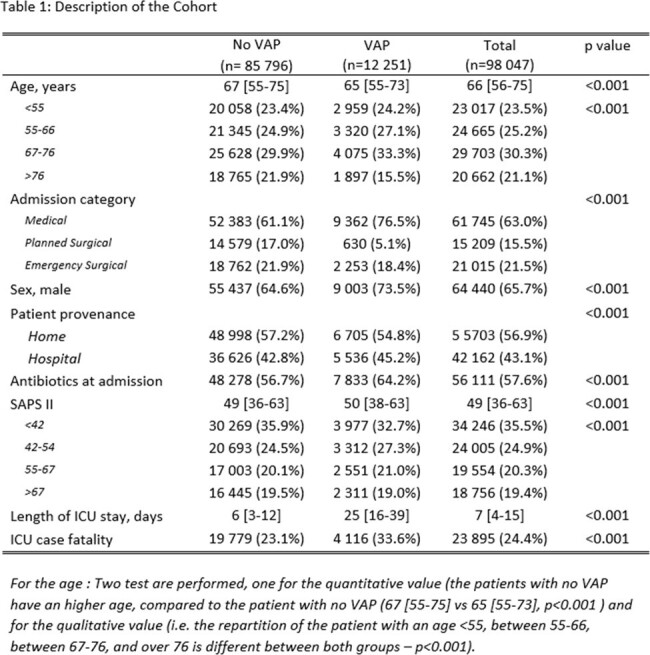

**Results:**

In this cohort, 98 047 patients were included. Among them, 12 251 (12.5%) had a VAP; the incidence rate for 1000 ventilation days was 18.9 cases per 1000 ventilation days. The median age of the cohort is 66 [55-75] years old with a male predominance (63.0 %). The SAPS score is 49 [36-63], the mortality is 24.4 % and the median ICU length of stay is 7 [4-15] days. Ventilated patients are more frequently admitted for medical (63.0 %) compared to surgical (37.0%) reason.

At day 30, the attributable mortality associated with ventilator-associated pneumonia (VAP) was estimated at 4.9% (3.7% - 6.2%). Out of 15,575 diagnosed cases of VAP, there were 4,695 instances of polymicrobial VAP, which constitutes 30.1% of the cases. Non-fermenting Gram-negative bacteria were a significant group, contributing to 25.4% of the VAP cases, with Pseudomonas aeruginosa being the most significant contributor (3 924 cases, 76.2% of non-fermenters)

Description of the microbial ecology of VAP

**Figure:**
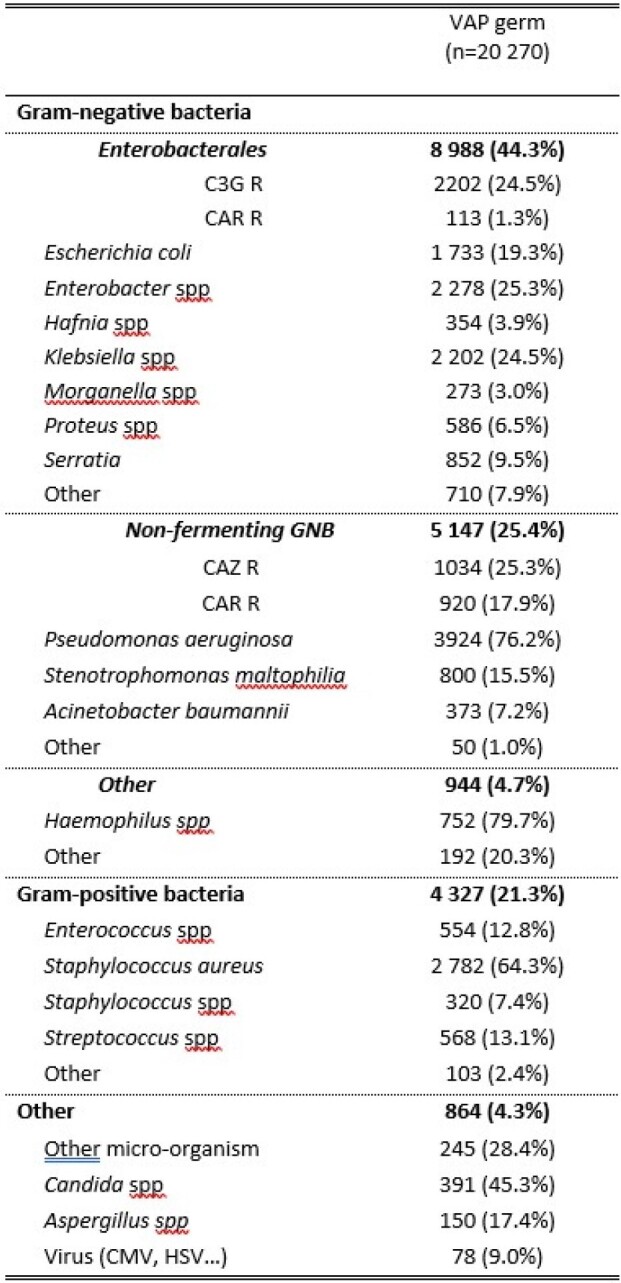

**Conclusion:**

The development of VAP is frequent, especially in patients admitted for medical reasons, with high severity and appears to be associated with an increased use of medical devices, like urinary and central venous catheters, reflecting the complexity of their clinical management.

**Disclosures:**

**Xavier Bourge, PharmD**, MSD France: Employee **Arnaud Friggeri, MD**, MSD France: Advisor/Consultant **Alain Lepape, MD**, MSD France: Grant/Research Support **Claire Prevot, n/a**, MSD France: Employee **Anais Machut, n/a**, MSD France: Grant/Research Support **Charles-Hervé Vacheron, MD**, MSD France: Grant/Research Support

